# The Radiographic Characteristics of Mandibular Wisdom Teeth That Can Cause Severe Deep Neck Infection

**DOI:** 10.7759/cureus.70791

**Published:** 2024-10-03

**Authors:** Eiji Iwata, Junya Kusumoto, Takumi Hasegawa, Akira Tachibana, Masaya Akashi

**Affiliations:** 1 Oral and Maxillofacial Surgery, Kakogawa Central City Hospital, Kakogawa, JPN; 2 Oral and Maxillofacial Surgery, Kobe University Hospital, Kobe, JPN

**Keywords:** deep neck abscess, deep neck infections, dentigerous cyst, mandibular wisdom tooth, necrotizing soft tissue infection, radicular cyst

## Abstract

Purpose: Mandibular wisdom teeth can occasionally cause infections, which can progress to severe deep neck infections (DNIs) including deep neck abscesses or necrotizing soft tissue infections, which are fatal. This study aimed to identify the radiographic characteristics of mandibular wisdom teeth that developed severe DNIs.

Methods: This study included patients who were admitted for the treatment of severe mandibular wisdom tooth infection between July 2012 and June 2024 at a single center. Patient characteristics, clinical data, and radiographic findings were analyzed and compared between the severe DNI group and mild DNI group including patients with cellulitis or superficial abscess. *P* < 0.05 was considered significant.

Results: Nineteen of 42 patients (45.2%) were included in the severe DNI group. The multivariate analysis showed that the highest odds ratio (OR) was for the presence of a radicular cyst (OR=17.7), followed by the presence of a dentigerous cyst (OR =14.5). The most common mandibular wisdom tooth with a dentigerous cyst in patients with severe DNIs was inverted according to Winter’s classification and type IIIC in the Pell and Gregory classification.

Conclusion: Radiographic characteristics associated with severe DNIs included the presence of radicular and dentigerous cysts in the mandibular wisdom teeth. Especially in dentigerous cysts, deeply impacted teeth should be taken attention.

## Introduction

Although the prevalence of deep neck infections (DNIs) has decreased with the spread of antibiotics, it remains a significant problem [1]. A previous study using the United Kingdom’s National Health Service data reported annual rates were from 0.55 to 2.45 per 100,000 for DNIs [2]. DNIs are accompanied by challenges in diagnosis and treatment and may lead to life-threatening complications such as descending necrotizing mediastinitis, septic shock, and pleural and pericardial effusion [3]. The infection typically spreads along the fascial planes and neck spaces [4]. The clinical symptoms of DNIs depend on the infected neck space. Common manifestations include pain, fever, swelling, dysphagia, trismus, and dyspnea, which occasionally result in fatality [4]. Blood tests are necessary to define the extent and severity of the condition. In addition to non-specific markers of inflammation such as the white blood cell (WBC) count, neutrophil count, and C-reactive protein (CRP), specific blood tests such as procalcitonin and delta neutrophil index can be used [5]. Among DNIs, necrotizing soft tissue infections (NSTI) and deep neck abscesses (DNA) are rare and fatal. They require early detection and surgical treatment, including drainage and debridement [6]. We previously reported diagnostic tools for NSTI in the oro-cervical region [7,8]. It goes without saying that although early diagnosis is important to save lives, preventing its occurrence is more valuable.

Odontogenic infections are the most common cause of DNIs, accounting for approximately 43% of all cases [9]. In odontogenic infections, mandibular wisdom teeth are one of the most common causes of DNIs, while odontogenic abscess in the lower jaw is caused predominantly by the first mandibular molars, followed by the premolars, the second molars and then the wisdom teeth [10,11]. Mandibular wisdom teeth tend to be partially erupted, which increases the risk of acute local soft tissue infections, including pericoronitis and periodontitis [12]. Owing to the associated spectrum of symptoms, ranging from mild pain to severe infection with trismus or difficulty in breathing, it is useful to investigate the characteristics of mandibular wisdom teeth with higher risks of producing pathologies. The management of mandibular wisdom teeth, including the decision to extract them, is often accompanied by confusion and controversy. To the best of our knowledge, no studies have investigated the radiographic characteristics of mandibular wisdom teeth which can cause DNIs, especially severe DNIs including NSTI or DNA. Radiographic characteristics of mandibular wisdom teeth include presence of cyst, angulation (tooth axis), impaction status, and overlap between the tooth root and the mandibular canal [13]. Therefore, the objective of the present study was to identify the radiographic characteristics of mandibular wisdom teeth that developed severe DNIs (NSTI or DNA) by comparing them with those of teeth that developed mild DNIs.

## Materials and methods

Patients

This study included patients admitted for the treatment of severe mandibular wisdom tooth infection between July 2012 and June 2024 at Kakogawa Central City Hospital. The following inclusion criteria were set: both sexes, over 18 years of age, and hospitalized for treatment with intravenous antibiotics for over 48 hours. The following hospitalization criteria were set: clinical findings such as skin erythema, dysphagia, difficulty eating, and high inflammation in blood tests [7,8]. The exclusion criteria were as follows: patients who were transferred to other hospitals for some reason, patients who underwent DNIs after extraction of the mandibular wisdom tooth, and patients who did not wish to participate after the publication of this study. The patients were divided into two groups: severe DNIs group and mild DNIs group. The severe DNIs group included patients who were diagnosed with DNA or NSTI, which are severe and lethal. The definition of NSTI was based on previous studies [7,8]. Briefly, they were diagnosed based on Fisher’s [14] and Mathieu’s [15] diagnostic criteria and confirmed by evidence of gas production on computed tomography (CT) images, intraoperative findings, and histopathology. The mild DNIs group included patients with cellulitis or superficial abscess (local onset, no spread into the deep anatomical space).

Data collection

**Figure 1:**
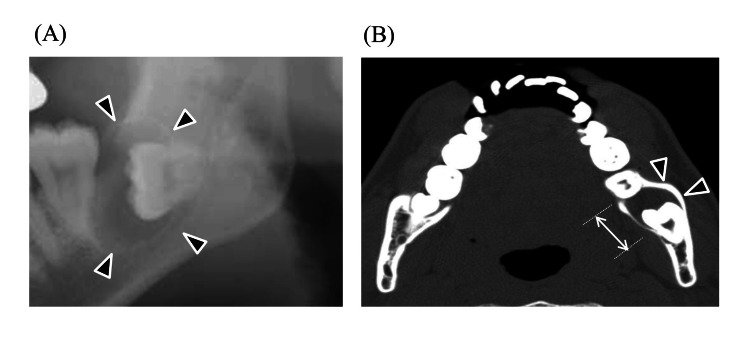
Dentigerous cyst (A) Panoramic image and (B) axial CT image. The maximum diameter was measured by the length of white arrow. Black arrows indicate the part of a dentigerous cyst.

**Figure 2:**
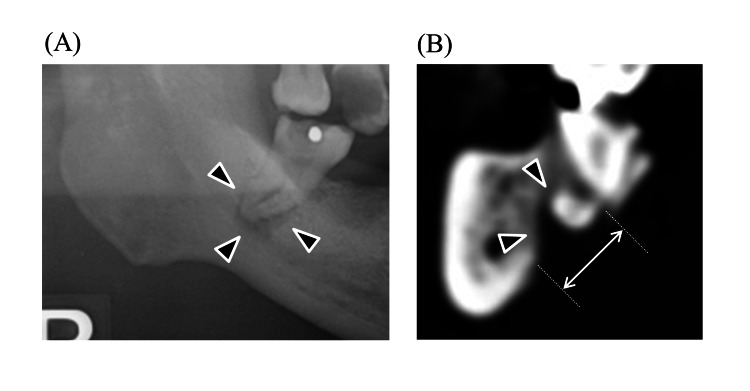
Apical radiolucency (A) Panoramic image and (B) MPR image. The maximum diameter was measured by the length of white arrow. Black arrows indicate the part of an apical radiolucency.

**Figure 3:**
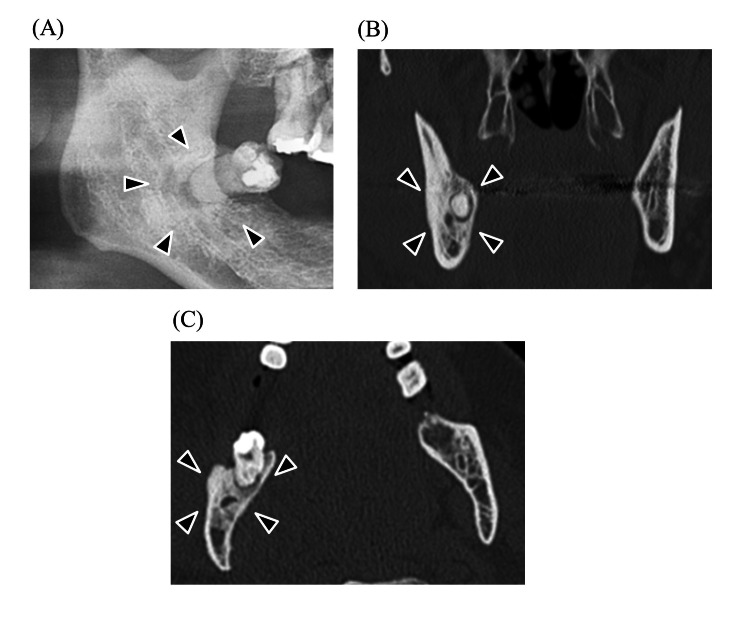
Radiopaque change in bone surrounding tooth (A) Panoramic image, (B) coronal CT image, and (C) axial CT image. Black arrows indicate the part of a radiopaque change in bone surrounding tooth.

The following variables from the medical records were retrospectively reviewed and investigated: patient age, sex, compromised host, side, angulation, horizontal position, vertical position, presence of pericoronal radiolucency, presence of apical radiolucency, presence of radiopaque changes in the surrounding bone, inflammatory markers in blood tests (CRP and WBC count), hospitalization duration, and outcome. All exams, including radiographic and blood tests, were done at the time of admission. The compromised host was defined as a patient with the following diseases, e.g., rheumatoid arthritis, kidney failure, and diabetes. Angulation was analyzed using a panoramic image based on Winter’s classification [16]. Briefly, vertical is the long axis of the mandibular wisdom tooth parallel to the mandibular second molar, mesioangular is the long axis of the mandibular wisdom tooth inclined in the mesial direction to the mandibular second molar, horizontal is the long axis of the mandibular wisdom tooth perpendicular to the mandibular second molar, distoangular is the long axis of the mandibular wisdom tooth inclined in the distal direction to the mandibular second molar, and inverted is the crown of the mandibular wisdom tooth directed to the basilar of the mandible [16]. The horizontal position was analyzed using a panoramic image based on the Pell and Gregory (PG) classification [17-19]. Briefly, Class I refers to a condition in which the distance between the distal surface of the mandibular third molar and the anterior margin of the mandibular ramus is greater than the anteroposterior dimension of the crown of the mandibular third molar. In Class II, the distance between the distal surface of the mandibular second molar and the anterior margin of the mandibular ramus was smaller than the anteroposterior dimension of the crown of the mandibular third molar. Class III refers to a condition in which there is no space between the distal surface of the second molar and the anterior margin of the mandibular ramus. Furthermore, the vertical position was investigated using a panoramic image based on the PG classification [17-19]. Position A refers to the occlusal plane of the mandibular third molar lying at or above the occlusal plane of the adjacent mandibular second molar. Position B refers to the condition in which the occlusal plane lies somewhere between the occlusal line and the cemento-enamel junction (CEJ) of the mandibular second molar. Position C refers to the condition in which the mandibular third molar was located below the CEJ of the adjacent mandibular second molar. When missing mandibular second molar, the last tooth was substituted. Furthermore, two odontogenic lesions were investigated using panoramic images and CT images. If pericoronal radiolucency was present, the maximum diameter of the cyst was measured on axial CT image (Figure 1). A dentigerous cyst was defined as pericoronal translucency ≥2.5 mm with reference to a previous study [20]. If an apical radiolucency was present, the maximum diameter of the cyst was measured on multi-planar reconstruction (MPR) image (Figure 2). A radicular cyst was defined as an apical radiolucency of ≥5 mm with reference to a previous study [21]. In addition, radiopaque changes in the bone surrounding the tooth were defined as an apparent increase in radiopacity around the tooth compared to the same position on the contralateral side on panoramic images and CT images (Figure 3). All CT images were acquired using a 64-slice CT system (Aquilion 64; Canon Medical Systems Corp., Tochigi, Japan) or a 128-slice CT system (SOMATOM Definition Flash; Siemens, Munich, Germany). Data were acquired under typical head and neck CT scanning conditions (120 kV, 1-5 mm slice) with automatic exposure control. Based on CT scans, Digital Imaging and Communications in Medicine viewer software (Zyostation2 TypeH; Ziosoft Inc., Tokyo, Japan) was used to prepare MPR sections. The contrast media used included Iomeron 300 (Eisai, Tokyo, Japan), Iopamirdol 370 (Hikari Pharmaceutical, Tokyo, Japan), and Omnipaque 300 (GE Healthcare, Chicago, IL, USA).

Ethical approval

This study was conducted according to the 1964 Declaration of Helsinki guidelines. Ethical approval was obtained from the Institutional Review Board (IRB) of Kakogawa Central City Hospital (authorization number: 2019-85). The ethics committee approved this study and provided administrative permission to access the data used in this study. As this was a retrospective study, the research plan was published on the homepages of the participating hospitals according to the instructions of the IRB following the guaranteed opt-out opportunity.

Statistical analysis

All statistical analyses were performed using SPSS version 26.0 (IBM Corp., Armonk, NY, USA). A receiver operating characteristic (ROC) curve was used to determine the cutoff values for age. The area under the ROC curve (AUC) was used to measure the discrimination accuracy. The association of each variable with severe DNIs was analyzed using the Mann-Whitney U test for ordinal variables and Fisher’s exact test or chi-square test for categorical variables. Statistical significance was set at *P* < 0.05. All variables associated with severe DNIs were introduced into a multiple logistic regression model. Before introduction, a multicollinearity test was performed to reject variables that did not fit the model significantly. Moreover, after multiple logistic regression, a goodness-of-fit analysis was performed. The Variance Inflation Factor (VIF) was used to determine the presence or absence of multicollinearity among factors. Odds ratios (OR) and 95% confidence intervals (CIs) were determined.

## Results

**Figure 4:**
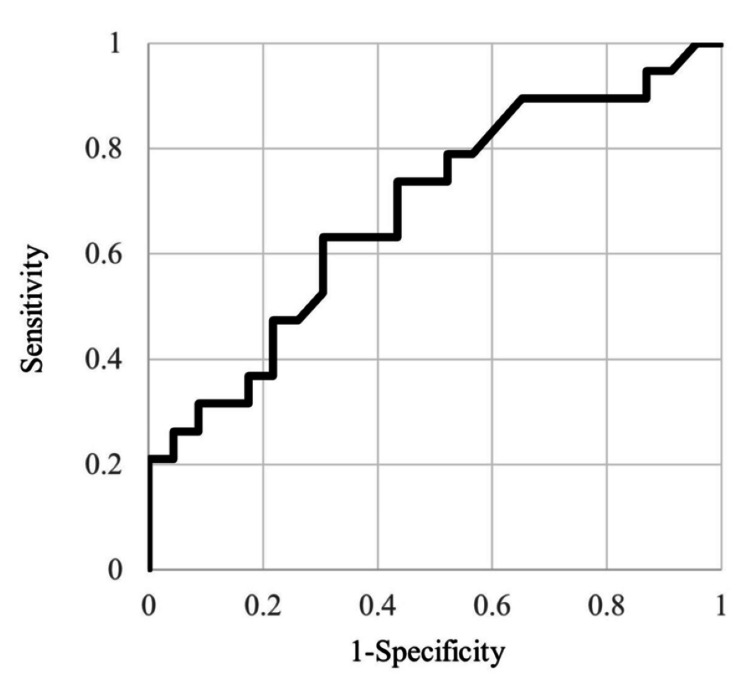
The receiver operating characteristic (ROC) curve for accuracy of age The area under the curve (AUC) for our model was 0.682 (95% confidence interval 0.516 to 0.848).

Nineteen of 42 patients (45.2%) were included in the severe DNIs group (Table 1). The remaining 23 patients were assigned to the mild DNIs group. Age ≥57 had a sensitivity of 63.2%, a specificity of 69.7%, and an AUC of 0.682 (Figure 4). Univariate analysis showed that age, angulation, vertical position, apical radiolucency, and radiopaque changes in the bone surrounding the tooth were significantly correlated with severe DNIs (Table 1). Patients with severe DNIs had higher CRP levels and longer hospitalization durations than those with mild DNIs. Most patients were rescued by antibiotic administration and surgical drainage, including debridement of necrotic tissues; however, one patient died.

**Table 1:** Results of univariate analysis of risk factors for severe deep neck infections (DNIs) ^a^Mann-Whiteny U test. ^b^Fisher’s exact test. ^c^Chi-squared test. * *P* < .05. CRP: C-reactive protein; WBC: white blood cell; Winter: Winter’s classification; PG: Pell and Gregory classification.

Variable		Severe DNIs group (n=19)	Mild DNIs group (n=23)	*P* value
Age (years)	Median (range)	60.0 (23-84)	42.0 (19-76)	0.041*^a^
Sex	Male	12 (63.2%)	12 (52.2%)	0.542^b^
	Female	7 (36.8%)	11 (47.8%)	
Compromised host	Yes	2 (10.5%)	2 (8.7%)	1.000^b^
	No	17 (89.5%)	21 (91.3%)	
Side	Left	8 (42.1%)	9 (39.1%)	1.000^b^
	Right	11 (57.9%)	14 (60.9%)	
Angulation (Winter)	Vertical	0 (0.0%)	3 (13.0%)	0.013*^c^
	Mesioangular	8 (42.1%)	10 (43.5%)	
	Horizontal	6 (31.6%)	3 (13.0%)	
	Distoangular	0 (0.0%)	6 (26.1%)	
	Inverted	5 (26.3%)	1 (4.3%)	
Horizontal position (PG)	Class I	7 (36.8%)	16 (69.6%)	0.071^c^
	Class II	6 (31.6%)	5 (21.7%)	
	Class III	6 (31.6%)	2 (8.7%)	
Vertical position (PG)	Position A	10 (52.6%)	21 (91.4%)	0.017*^c^
	Position B	3 (15.8%)	1 (4.3%)	
	Position C	6 (31.6%)	1 (4.3%)	
Peri-coronal radiolucency	No	8 (42.1%)	14 (60.9%)	0.105^c^
	<2.5mm	2 (10.5%)	5 (21.7%)	
	≥2.5mm (Dentigerous cyst)	9 (47.4%)	4 (17.4%)	
Apical radiolucency	No	13 (68.4%)	17 (73.9%)	0.045*^c^
	<5mm	0 (0.0%)	4 (17.4%)	
	≥5mm (Radicular cyst)	6 (31.6%)	2 (8.7%)	
Radiopaque change in bone surrounding tooth	Yes	13 (68.4%)	7 (30.4%)	
	No	6 (31.6%)	16 (69.6%)	0.028*^b^
CRP (mg/dL)	Median (range)	13.4 (5.9-40.7)	5.6 (0.5-20.1)	<0.001*^a^
WBC (10^3^/uL)	Median (range)	13.7 (6.9-21.2)	11.5 (7.1-24.9)	0.308^a^
Hospitalization duration (days)	Median (range)	14.0 (7-34)	7.0 (3-12)	<0.001*^a^
Outcome	Survival	18 (94.7%)	23 (100.0%)	0.452^b^
	Mortality	1 (5.3%)	0 (0.0%)	

Before introducing the multiple logistic regression model, a multicollinearity test was performed using the VIF. As a result, the VIF value was as low as less than 6, and no multicollinearity was observed. Therefore, the other variables were not excluded. The multivariate analysis showed that the highest OR was for the presence of a radicular cyst (OR=17.7), followed by the presence of a dentigerous cyst (OR =14.5) (Table 2). After multivariate analysis, goodness-of-fit analysis was performed using the Hosmer-Lemeshow test. The values were 0.324. This indicates no problem with the model's goodness of fit.

**Table 2:** Results of multivariate analysis of risk factors for severe deep neck infections (DNIs) ^* ^*P* < .05

Variable	*P* value	Odds ratio	95% CI	
			Lower	Upper
Age ≥57 years (vs. <57 years)	0.704	1.560	0.157	15.511
Female (vs. Male)	0.675	0.663	0.097	4.537
Compromised host (vs. No)	0.402	0.311	0.020	4.761
Right (vs. Left)	0.918	0.906	0.137	5.992
Inverted (vs. Others)	0.677	3.741	0.008	1862.762
Class III (vs. Class I/II)	0.940	0.787	0.002	396.581
Position C (vs. Position A/B)	0.560	4.629	0.027	800.317
Dentigerous cyst (vs. No/<2.5mm)	0.012*	14.503	1.799	116.918
Radicular cyst (vs. No/<5mm)	0.025*	17.655	1.432	217.662
Radiopaque change in bone surrounding tooth (vs. No)	0.672	1.587	0.188	13.427

Table 3 shows differences in characteristics and radiographic findings in groups with or without radiopaque change in the bone surrounding the tooth. Although age, angulation, horizontal position, vertical position, and apical radiolucency were significantly related to the radiopaque change in the bone surrounding the tooth, peri-coronal radiolucency was not associated.

**Table 3:** Differences of characteristics and radiographic findings in groups with or without radiopaque change in bone surrounding tooth ^a^Mann-Whiteny U test. ^b^Chi-squared test. ^* ^*P* < .05 Winter: Winter’s classification; PG: Pell and Gregory classification.

Variable		Radiopaque change in bone surrounding tooth group (n=20)	Non-radiopaque change in bone surrounding tooth group (n=22)	*P* value
Age (years)	Median (range)	64.5 (24-84)	36.0 (19-76)	0.003*^a^
Angulation (Winter)	Vertical	2 (10.0%)	1 (4.5%)	0.032*^b^
	Mesioangular	7 (35.0%)	11 (50.0%)	
	Horizontal	7 (35.0%)	2 (9.1%)	
	Distoangular	0 (0.0%)	6 (27.3%)	
	Inverted	4 (20.0%)	2 (9.1%)	
Horizontal position (PG)	Class I	14 (70.0%)	17 (77.3%)	0.020*^b^
	Class II	0 (0.0%)	4 (18.2%)	
	Class III	6 (30.0%)	1 (4.5%)	
Vertical position (PG)	Position A	14 (70.0%)	17 (77.3%)	0.020*^b^
	Position B	0 (0.0%)	4 (18.2%)	
	Position C	6 (30.0%)	1 (4.5%)	
Peri-coronal radiolucency	No	11 (55.0%)	11 (50.0%)	0.523^b^
	<2.5mm	2 (10.0%)	5 (22.7%)	
	≥2.5mm (Dentigerous cyst)	7 (35.0%)	6 (27.3%)	
Apical radiolucency	No	11 (55.0%)	19 (86.4%)	0.038*^b^
	<5mm	2 (10.0%)	2 (9.1%)	
	≥5mm (Radicular cyst)	7 (35.0%)	1 (4.5%)	

**Figure 5:**
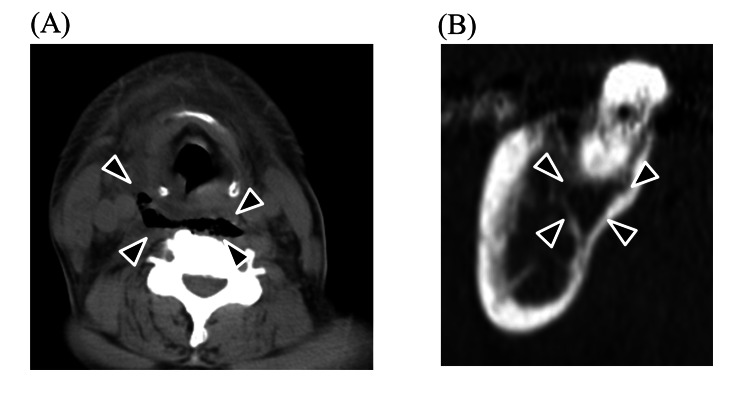
CT images at first visit of a patient (No.17) (A) Axial CT image. Black arrows indicate the part of a gas production in the retropharyngeal space. (B) MPR image. Black arrows indicate the part of a radicular cyst.

**Figure 6:**
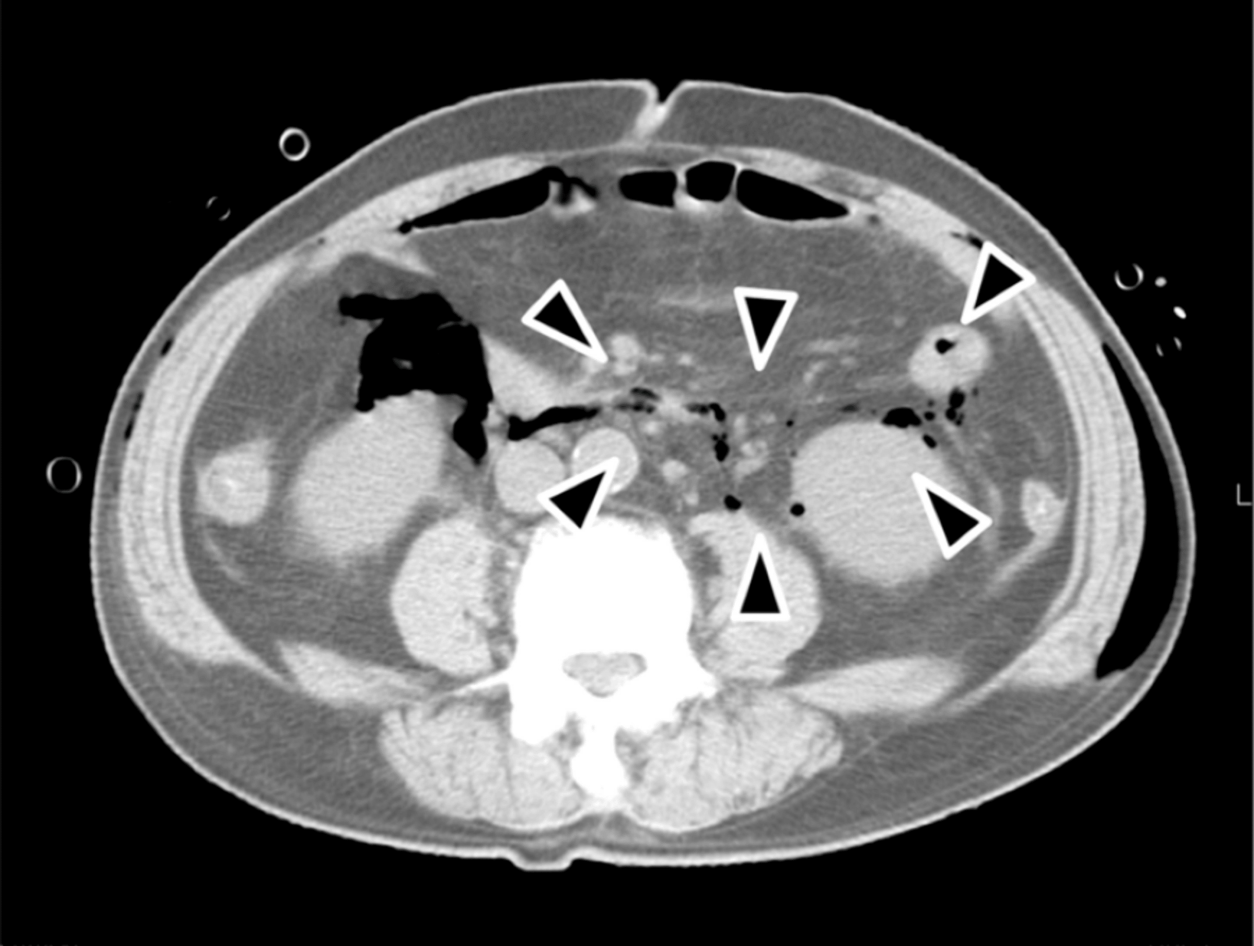
CT image under hospitalization of a patient (No.17) Axial CT image. Black arrows indicate the part of a gas production in the abdominal cavity.

Table 4 shows the patients' characteristics, clinical data, and radiographic findings in the severe DNIs group. The most common mandibular wisdom tooth with a radicular cyst was mesioangular according to the Winter’s classification (four of six patients [66.7%]) and type IA in the PG classification (five of six patients [83.3%]). On the other hand, the most common mandibular wisdom tooth with a dentigerous cyst was inverted in the Winter’s classification (four of nine patients [44.4%]) and type IIIC in the PG classification (three of nine patients [33.3%]). Three of 19 patients (15.8%) had NSTI, and only one patient (No. 17) died. He visited our hospital with swelling and erythema extending from the left side of the face to the neck and difficulty in breathing. Blood test detected higher levels of inflammatory markers (CRP: 40.7 mg/dL, WBC: 19100/uL) (Table 4). He produced gas in various neck spaces, including the retropharyngeal space (Figure 5A). The cause was an infection of the mandibular wisdom tooth with a radicular cyst but did not have radiopaque changes in the bone surrounding the tooth (Figure 5B). On the same day, an emergency operation including drainage under general anesthesia was performed, but the airway became blocked during the operation, and the patient's heart and lungs stopped without being able to secure the airway. After cardiac massage for 20 minutes and emergency tracheotomy, return of circulation was observed. However, the anesthesiologists determined that continuing the surgery would be life-threatening and so the operation was finished before drainage. After surgery he was admitted to the intensive care unit and underwent multimodal therapy including antibiotic therapy. However, the spread of the infection could not be controlled and he produced gas spread to the abdominal cavity on the next day (Figure 6). Unfortunately, he died of multiple organ failure due to sepsis and hypoxic encephalopathy on the eighth day of hospitalization.

**Table 4:** Characteristics, clinical data, and radiographic findings of patients in severe deep neck infections (DNIs) group DNA: deep neck abscess; NSTI: necrotizing soft tissue infection; CRP: C-reactive protein; WBC: white blood cell; DM: Diabetes mellitus; Winter: Winter’s classification; PG: Pell and Gregory classification.

No.	Age	Sex	Compromised host	Winter	PG	Peri-coronal radiolucency	Apical radiolucency	Radiopaque change in bone surrounding tooth	CRP (mg/dL)	WBC (10^3^/uL)	Diagnosis	Outcome
1	38	M	-	Horizontal	IA	-	Radicular cyst	+	12.2	18.4	DNA	Survival
2	84	F	-	Inverted	IIIC	<2.5mm	-	+	9.8	15.7	DNA	Survival
3	80	M	-	Mesioangular	IIC	Dentigerous cyst	-	+	12.9	13.7	DNA	Survival
4	83	F	-	Inverted	IIIC	Dentigerous cyst	-	+	8.8	13.0	DNA	Survival
5	60	M	-	Horizontal	IIA	<2.5mm	-	+	16.3	6.9	DNA	Survival
6	36	M	-	Horizontal	IIB	-	-	-	17.0	17.0	DNA	Survival
7	46	M	-	Mesioangular	IA	-	Radicular cyst	+	10.7	9.4	DNA	Survival
8	34	M	-	Horizontal	IA	Dentigerous cyst	-	-	12.3	21.2	DNA	Survival
9	73	F	DM	Mesioangular	IIB	Dentigerous cyst	-	-	15.6	14.4	DNA	Survival
10	64	M	DM	Horizontal	IIA	Dentigerous cyst	-	+	13.7	10.0	DNA	Survival
11	23	M	-	Mesioangular	IA	-	-	-	13.4	16.5	DNA	Survival
12	57	F	-	Horizontal	IIA	Dentigerous cyst	-	+	19.2	13.4	DNA	Survival
13	79	F	-	Horizontal	IIIC	-	Radicular cyst	+	11.4	10.8	DNA	Survival
14	50	F	-	Mesioangular	IA	-	Radicular cyst	+	20.8	8.8	DNA	Survival
15	67	M	-	Inverted	IIIB	Dentigerous cyst	-	-	22.0	10.1	DNA	Survival
16	65	F	-	Inverted	IIIC	Dentigerous cyst	-	+	12.0	11.3	DNA	Survival
17	59	M	-	Mesioangular	IA	-	Radicular cyst	-	40.7	19.1	NSTI	Mortality
18	70	M	-	Inverted	IIIC	Dentigerous cyst	-	+	28.9	16.9	NSTI	Survival
19	25	M	-	Mesioangular	IA	-	Radicular cyst	+	5.9	14.3	NSTI	Survival

## Discussion

This study aimed to identify the radiographic characteristics of mandibular wisdom teeth that develop severe DNIs. Nineteen of 42 patients (45.2%) had DNA or NSTI. The multivariate analysis showed that the highest OR was observed for a radicular cyst (OR=17.7), followed by a dentigerous cyst (OR=14.5). Most patients with severe DNIs were rescued by antibiotic administration and surgical drainage, including debridement of necrotic tissues; however, one patient died from NSTI caused by an infection of the mandibular wisdom tooth with a radicular cyst.

In this study, we found that the presence of radicular and dentigerous cysts in mandibular wisdom teeth is an independent risk factor for severe DNIs. Around the mandibular wisdom, teeth tend to have poor hygiene. This is because of their posterior location and tendency to erupt partially; these teeth are technically difficult to clean [22]. This is why food traps develop, which are difficult to clean, making teeth susceptible to caries and periodontitis [22]. Inflammation extends over the distal occlusal surface and harbors bacteria, resulting in pericoronitis. Mandibular wisdom teeth have enamel fissures and deep grooves susceptible to caries [22]. Infection from caries spreads into the periapical area, where bacterial endotoxins trigger epithelial cells to multiply and occasionally progress to radicular cysts [22]. Among all jaw cysts, radicular cysts make up approximately 52-68%, and their incidence is highest in the third and fourth decades of life, with male predominance [21]. Radicular cysts are usually asymptomatic and detected incidentally on panoramic images while investigating other diseases. However, once infected, it causes pain and swelling, after which the patient becomes aware of the problem. On the other hand, dentigerous cysts are epithelial-lined developmental cysts surrounding the crown of an unerupted or impacted tooth and account for approximately 24% of the epithelium-lined cysts of the jaw [23]. The most common tooth location is the mandibular wisdom teeth, probably because the long duration of impaction increases exposure to inflammatory stimuli. Dentigerous cysts are benign and generally slow-growing, but they can become problematic when infected. They are usually asymptomatic unless accompanied by secondary inflammation including pericoronitis and marginal or apical periodontitis of adjacent teeth [24,25]. When a dentigerous cyst becomes secondarily infected, it can lead to symptoms such as pain, swelling, and discomfort [26]. The infection can cause the cyst to enlarge more rapidly, potentially leading to the displacement of adjacent teeth or even resorption of the roots of nearby teeth [27]. Therefore, cysts in either disease mean presence of large lesions with inflammation and cause resorption of jawbone which can lead to the spread of infection to neck spaces (Figure 1B, 2B).

As radiographic characteristics of mandibular wisdom tooth, angulation (Winter’s classification) and impaction status (PG classification) are well known, besides overlap between the tooth root and the mandibular canal [13,16-19]. Therefore, we furthermore investigated tendencies of these radiographic findings in mandibular wisdom teeth with cysts. Interestingly, the most common mandibular wisdom tooth with a radicular cyst in patients with severe DNIs was mesioangular according to Winter’s classification and type IA in the PG classification. On the other hand, the mandibular wisdom tooth with a dentigerous cyst in patients with severe DNIs was more likely to be inverted according to Winter's classification and type IIIC in the PG classification. This difference may be because of difference in lesion site as a radicular cyst is apical and a dentigerous cyst is peri-coronal. The former may be because a food trap between the mandibular second molar and wisdom tooth is developed by the mesioangular and larger crown area is exposed in the oral cavity by type IA, resulting in a higher risk of developing caries. The latter is discussed as follows.

We also focused on radiopaque changes in the bone surrounding the tooth. A recent review showed that radiopaque changes in the bone surrounding the tooth indicated a sclerosing osteolitis response to long-standing chronic apical infection [28]. It accounts for approximately 7% of all periapical radiolucencies with inflammation and is seen much more frequently in the mandible than in the maxilla [28]. Especially, it appears as a dense radiopaque change around the apices of teeth infected with necrotic pulp. It histologically shows the replacement of cancellous bone with compact bone and a viable inflammatory reaction [28]. In the present study, age, angulation, horizontal position, vertical position, and apical radiolucency were significantly related to radiopaque change in the bone surrounding the tooth. Among them, inverted, class III, and position C, besides radicular cyst (apical radiolucency) all mean that the lesion existed deep in the jawbone. Under these conditions, the formation of an anaerobic environment may be facilitated. It is well known that severe infections in oral and neck regions is mainly caused by anaerobic bacteria [29]. In addition, if the lesion exists deeply in the jawbone, it can spread infection to neck spaces including submandibular space directly. Those may explain why the most common mandibular wisdom tooth with a dentigerous cyst (which itself has no significant correlation with radiopaque changes in the surrounding bone) in patients with severe DNIs was inverted in the Winter’s classification and type IIIC in the PG classification. 　　　 

To the best of our knowledge, this is the first study to investigate the radiographic characteristics of mandibular wisdom teeth that develop severe DNIs. However, this study had some limitations. First, it was a retrospective study. There is the possibility of unknown confounding factors. Second, the population and number of outcomes evaluated in this study were small, which might have introduced bias in the data selection and analyses. Third, this study did not include details of the periodontal status, which influences the development of inflammation, including pericoronitis. Finally, this study did not include patients with infection of the mandibular wisdom tooth that did not progress to DNIs. However, we considered that the radiographic characteristics of mandibular wisdom teeth that developed severe DNIs are more evident by comparing with mild DNIs than comparing with infection which did not progress to DNIs. Prospective studies with larger sample sizes and more detailed examinations including periodontal examination and interview of history of pericoronitis should be conducted in the future.

## Conclusions

Radiographic characteristics associated with severe DNIs include the presence of radicular and dentigerous cysts in mandibular wisdom teeth, which indicate large lesions with inflammation that cause resorption of jawbone which can lead to the spread of infection to neck spaces. Especially in dentigerous cysts, deeply impacted teeth should be given attention, probably because the lesion that exists deeply in the jawbone can cause an anaerobic environment and spread infection to neck spaces including submandibular space directly.

In this study, one patient died from NSTI caused by an infection of the mandibular wisdom tooth with a radicular cyst. Dentists should recommend extraction by explaining the risks of developing severe DNIs if mandibular wisdom teeth with cysts are found on panoramic images.
